# Supplementary material for: Inhibition of Rice Stripe Virus Accumulation by Polyubiquitin-C in Laodelphax striatellus
Source: Insects. 2024 Feb 22;15(3):149. doi: 10.3390/insects15030149 (PMC10971706; doi:10.3390/insects15030149)
Supplement: Supplementary file 1 [file insects-15-00149-s001.zip › Supplementary Table S1.pdf]

Supplementary Table S1 Primers used in this study.

Primer name	Sequence (5' to 3')
RNAi-UBC-F	5'-TAATACGACTCACTATAGGGAGAGTTGACCG GCAAGACGATTAC-3'
RNAi-UBC-R	5'-TAATACGACTCACTATAGGGAGACCTTAACGT TCTCAATTGTG-3'
ORF-UBC-F	5'-ATGCAGATATTTGTGAAAAC-3'
ORF-UBC-R	5'-TTAGTCACCACGCATTCTTTC-3'
q-UBC-F	5'-CTGTCTGGAAATACTTTTAC-3'
q-UBC-R	5'-ACGCATTCTTTCAAGCAATC-3'
q-actin-F	5'-AATCGTAAGAGACATCAAGGAG-3'
q-actin-R	5'-AGGCAATTCGTAGGACTTCT-3'
q-RSV-NP-F	5'-TGCAGAAGGCAATCAATGACAT-3'
q-RSV-NP-R	5'-TGTCACCACCTTTGTCCTTCAA-3'
