# Supplementary figures and images for: Inhibition of Rice Stripe Virus Accumulation by Polyubiquitin-C in Laodelphax striatellus
Source: Insects. 2024 Feb 22;15(3):149. doi: 10.3390/insects15030149 (PMC10971706; doi:10.3390/insects15030149)

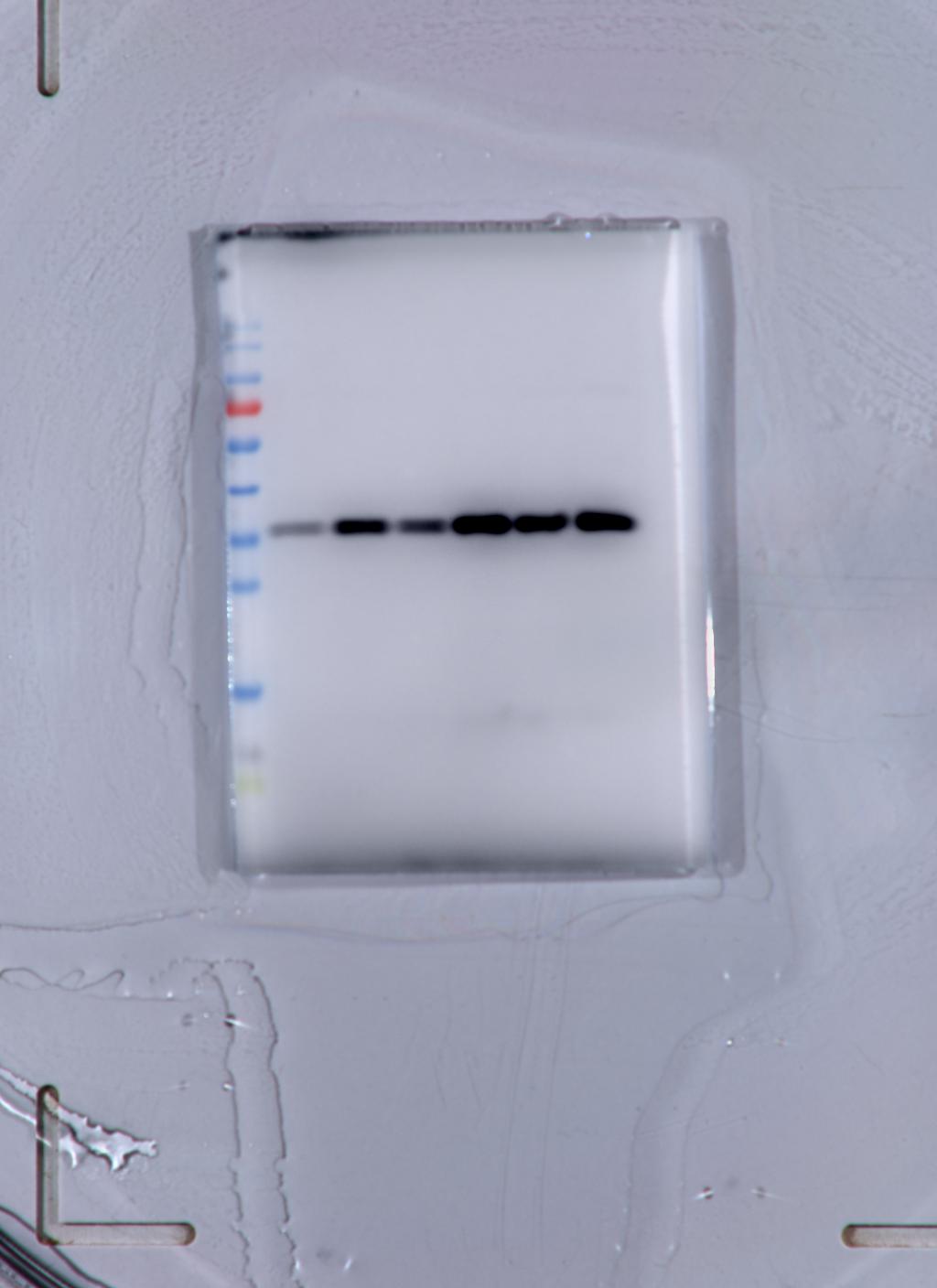

Supplement: Supplementary file 1 [file insects-15-00149-s001.zip › Figure S1. original western blot-1.jpg]

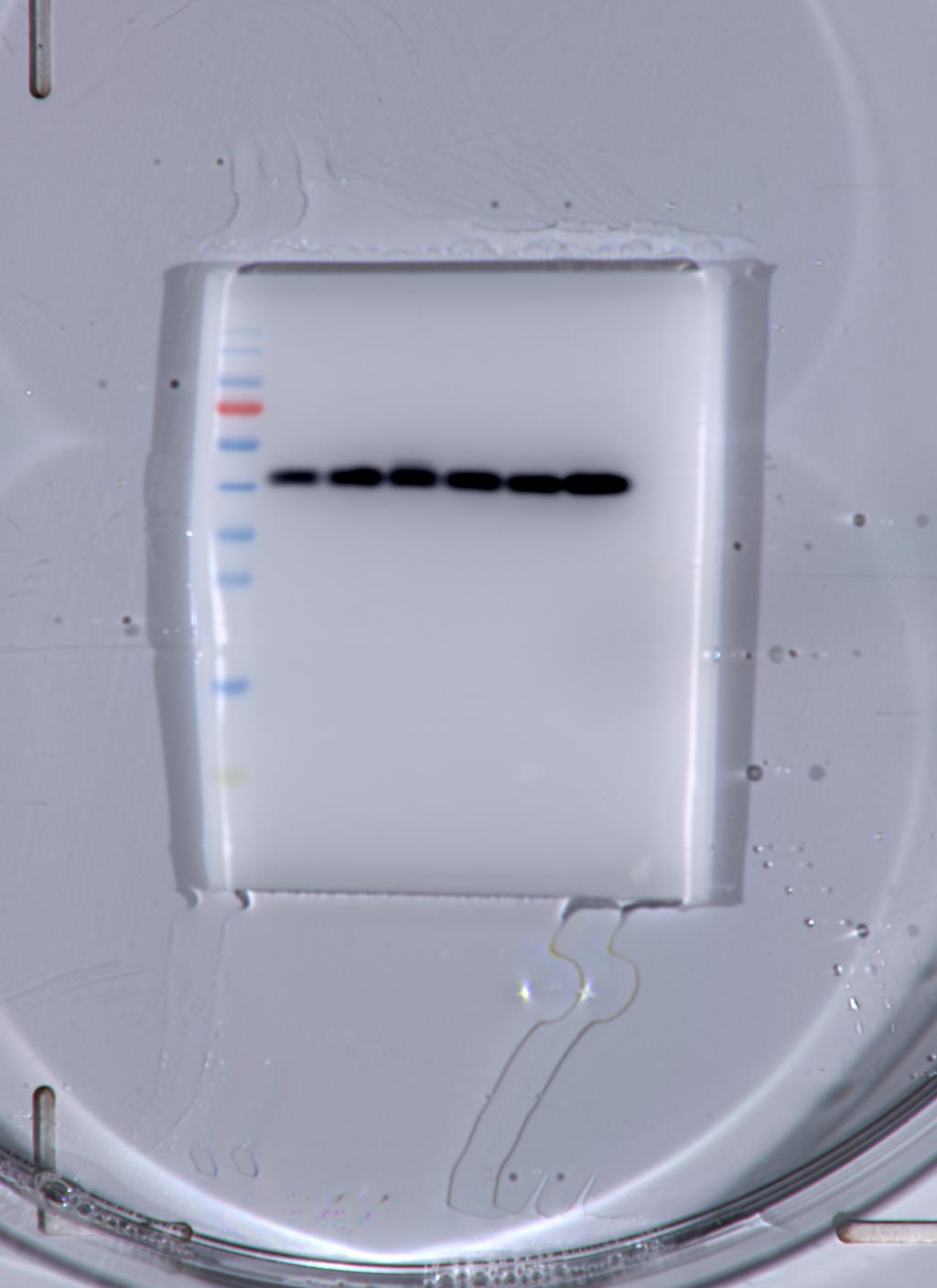

Supplement: Supplementary file 1 [file insects-15-00149-s001.zip › Figure S2. original western blot-2.jpg]
